# Supplementary material for: MetaRibo-Seq measures translation in microbiomes
Source: Nat Commun. 2020 Jun 29;11:3268. doi: 10.1038/s41467-020-17081-z (PMC7324362; doi:10.1038/s41467-020-17081-z)
Supplement: Supplementary file 10 — Supplementary Data 7 [file 41467_2020_17081_MOESM10_ESM.zip › File2/Confidence_High_Taxonomy/221197_out.krona.html]

Javascript must be enabled to view this page.

members
magnitude
magnitudeUnassigned
count
unassigned
taxon
rank

221197\_out

101

101
2
superkingdom

phylum
1239
100

100
class
186801

100
4

SRS018313\_contig\_number\_contig-100\_21089.54250SRS046712\_contig\_number\_contig-100\_15309.15310SRS075078\_contig\_number\_contig-100\_29938.112729SRS098881\_contig\_number\_18325
186802
order

6
541000
family

6
1263
genus
2

SRS049896\_contig\_number\_6852SRS057478\_contig\_number\_contig-100\_11216.11217

species
46228
4

SRS015264\_contig\_number\_contig-100\_3277.39684SRS020233\_contig\_number\_50766SRS052697\_contig\_number\_contig-100\_277.259107SRS148159\_contig\_number\_58695

186803
family

SRS013098\_contig\_number\_40021SRS016267\_contig\_number\_contig-100\_3093.80552SRS019030\_contig\_number\_contig-100\_28596.28596SRS097889\_contig\_number\_41760SRS098655\_contig\_number\_contig-100\_3157.42561SRS1041033\_contig\_number\_24177SRS104197\_contig\_number\_contig-100\_461.6620SRS143466\_contig\_number\_22369SRS147766\_contig\_number\_23412SRS148721\_contig\_number\_56312
10
90

841
genus
2

1
species
166486

SRS893270\_contig\_number\_contig-100\_9413.111229


SRS077502\_contig\_number\_15640
species
360807
1


SRS015217\_contig\_number\_contig-100\_2193.175871
1
species
2292902

2109690
species
1

SRS098644\_contig\_number\_29544

3
2292906
species

SRS013940\_contig\_number\_3062SRS024132\_contig\_number\_47901SRS146813\_contig\_number\_23935

21

SRS017103\_contig\_number\_30997SRS019068\_contig\_number\_114406SRS075878\_contig\_number\_7390SRS1041036\_contig\_number\_1339SRS144714\_contig\_number\_14523
5
572511
genus

418240
species
5

SRS014948\_contig\_number\_contig-100\_4133.52921SRS104311\_contig\_number\_44105SRS143780\_contig\_number\_33257SRS144362\_contig\_number\_18140SRS893259\_contig\_number\_7064

6
33038
species

SRS015431\_contig\_number\_89002SRS019601\_contig\_number\_contig-100\_422.169443SRS063518\_contig\_number\_39887SRS1055043\_contig\_number\_contig-100\_363.158474SRS146888\_contig\_number\_10527SRS971275\_contig\_number\_46807

2292976
species
1

SRS1041136\_contig\_number\_18046

species
33039
4

SRS014683\_contig\_number\_6270SRS015782\_contig\_number\_47508SRS020328\_contig\_number\_27052SRS042966\_contig\_number\_19489

3
207244
genus


SRS017916\_contig\_number\_9731SRS148511\_contig\_number\_15805SRS893358\_contig\_number\_contig-100\_14211.51938
649756
species
3

15
33042
genus

15
410072
species

SRS015890\_contig\_number\_8141SRS017433\_contig\_number\_contig-100\_5922.90939SRS018984\_contig\_number\_27630SRS019685\_contig\_number\_contig-100\_2014.144305SRS024331\_contig\_number\_3938SRS024492\_contig\_number\_8629SRS044535\_contig\_number\_contig-100\_9145.53535SRS045713\_contig\_number\_19738SRS048060\_contig\_number\_723SRS050422\_contig\_number\_contig-100\_997.202901SRS062654\_contig\_number\_contig-100\_19713.62478SRS1041091\_contig\_number\_22685SRS143181\_contig\_number\_11724SRS146812\_contig\_number\_63128SRS149181\_contig\_number\_contig-100\_15321.105214

genus
189330

SRS076804\_contig\_number\_contig-100\_9696.9697
1
34


SRS053356\_contig\_number\_45344SRS098514\_contig\_number\_contig-100\_280.4155SRS098827\_contig\_number\_contig-100\_7382.42356SRS143598\_contig\_number\_contig-100\_17374.17374
4
species
2293097


SRS042284\_contig\_number\_6270SRS143876\_contig\_number\_40923
2
2292347
species

12
39486
species

SRS013951\_contig\_number\_7298SRS014235\_contig\_number\_25711SRS015217\_contig\_number\_contig-100\_2179.175857SRS017191\_contig\_number\_contig-100\_6148.50866SRS017521\_contig\_number\_contig-100\_975.255139SRS056259\_contig\_number\_contig-100\_3221.173944SRS074586\_contig\_number\_contig-100\_49.40822SRS077392\_contig\_number\_40624SRS1041129\_contig\_number\_7803SRS1055067\_contig\_number\_14915SRS146764\_contig\_number\_31973SRS148091\_contig\_number\_309


SRS105153\_contig\_number\_contig-100\_343.189755SRS147022\_contig\_number\_5507SRS892882\_contig\_number\_contig-100\_41243.41244
3
species
2292346

88431
species
10

SRS011239\_contig\_number\_20367SRS014923\_contig\_number\_contig-100\_11165.140678SRS014979\_contig\_number\_27251SRS019161\_contig\_number\_25727SRS020869\_contig\_number\_38060SRS049712\_contig\_number\_24689SRS064276\_contig\_number\_29522SRS065504\_contig\_number\_36326SRS142503\_contig\_number\_27998SRS148196\_contig\_number\_29884

2
species
2292041

SRS058770\_contig\_number\_contig-100\_33616.73633SRS142890\_contig\_number\_contig-100\_29801.83733

1
phylum
976

200643
class
1

171549
order
1

family
171550
1

genus
239759
1


SRS063985\_contig\_number\_5530
1
species
328814
